# Development of Prevascularized Synthetic Block Graft for Maxillofacial Reconstruction

**DOI:** 10.3390/jfb16010018

**Published:** 2025-01-09

**Authors:** Borvornwut Buranawat, Abeer Shaalan, Devy F. Garna, Lucy Di Silvio

**Affiliations:** 1 Center for Implant Dentistry and Periodontics, Faculty of Dentistry and Research Unit in Innovations in Periodontics, Oral Surgery and Advanced Technology in Implant Dentistry, Thammasat University, Bangkok 10200, Thailand; borvornw@staff.tu.ac.th; 2 Center for Oral, Clinical and Translational Sciences, Faculty of Dentistry, Oral & Craniofacial Sciences, King’s College London, London SE1 9RT, UK; shaalan.abeer@kcl.ac.uk (A.S.); devy.garna@kcl.ac.uk (D.F.G.)

**Keywords:** prevascularization, porous calcium phosphate block graft, bone tissue engineering, growth factors

## Abstract

Cranio-maxillofacial bone reconstruction, especially for large defects, remains challenging. Synthetic biomimetic materials are emerging as alternatives to autogenous grafts. Tissue engineering aims to create natural tissue-mimicking materials, with calcium phosphate-based scaffolds showing promise for bone regeneration applications. This study developed a porous calcium metaphosphate (CMP) scaffold with physicochemical properties mimicking natural bone, aiming to create a prevascularized synthetic bone graft. The scaffold, fabricated using sintered monocalcium phosphate with poly (vinyl alcohol) as a porogen, exhibited pore sizes ranging from 0 to 400 μm, with the highest frequency between 80 and 100 μm. The co-culture of endothelial cells (ECs) with human alveolar osteoblasts (aHOBs) on the scaffold led to the formation of tube-like structures and intrinsic VEGF release, reaching 10,455.6 pg/mL This level approached the optimal dose for vascular formation. Conversely, the co-culture with mesenchymal stem cells did not yield similar results. Combining ECs and aHOBs in the CMP scaffold offers a promising approach to developing prevascularized grafts for cranio-maxillofacial reconstruction. This innovative strategy can potentially enhance vascularization in large tissue-engineered constructs, addressing a critical limitation in current bone regeneration techniques. The prevascularized synthetic bone graft developed in this study could significantly improve the success rate of maxillofacial reconstructions, offering a viable alternative to autogenous grafts.

## 1. Introduction

Cranio-maxillofacial bone defects, whether resulting from congenital deformities, trauma, disease, or cancer, pose significant clinical challenges, impacting not only physical function but also esthetics and psychological well-being. Approximately 2 million bone grafting procedures are performed globally each year, with 6% of these aimed at repairing cranio-maxillofacial defects [1]. Bone is a dynamic, highly vascularized tissue with regenerative and healing potential that is reliant on the close spatial and temporal connection of blood vessels and bone cells. Current treatments for bone repair typically involve autografts and allografts, which, despite being the gold standard, face challenges such as limited tissue availability, donor site morbidity, disease transmission risks, and potential immunogenic rejection [2,3]. The limitations of traditional transplanted grafts have sparked increased attention towards developing advanced synthetic bone graft materials over the past century, aiming to restore organ and tissue function [4,5].

Tissue engineering has evolved as an emerging therapy with the potential to replace or regenerate tissues or organs. This usually necessitates a scaffold to provide the structural architecture and act as a vehicle for incorporated factors to enhance the recruitment of stem cells. The importance of scaffolds has been highlighted by the current advances that are occurring in novel biomaterial design with bioactive potential, able to influence cell behaviour directly. However, large bone defects present a major hurdle: insufficient vascularization in the early stages post implantation, which limits nutrient and oxygen supply to the scaffold, leading to poor cell survival and integration. In vivo, blood vessels provide essential support to bone tissue, ensuring its regeneration and healing potential [6,7,8], but the supply of oxygen and nutrients is often limited to cells in a proximity of 100–200 µm from the next capillary [9]. In the case of large tissue-engineered constructs, the survival of cells in the centre cannot solely rely on nutrient supply by diffusion. Growth, function, and survival post implantation are entirely dependent on the ingrowth of blood vessels from the host [10]. Therefore, additional strategies that serve to enhance vascularization are essential for the survival of large tissue-engineered grafts.

An optimal scaffold for bone tissue engineering should exceed simple osteoconductive properties. It must actively stimulate osteoinduction while concurrently promoting angiogenesis and vascularization, thereby tackling the combined challenges of bone regeneration and adequate blood supply. An ideal scaffold for bone tissue engineering should mimic bone’s chemistry and structure, featuring high porosity (>40–60%) and interconnected pores. This design facilitates cell migration, nutrient exchange, and fluid transport, all crucial for cell survival and vascularization [11].

In vivo prevascularization, an early strategy to enhance vascularization, involves implanting the scaffold in a primary site for weeks to develop a microvascular network before reimplantation at the defect site. This method provides immediate vascularization through microsurgical attachment to local vasculature.

In vitro, prevascularization has emerged as a promising strategy to enhance scaffold performance and survival. This approach requires only one surgical procedure and reduces the time the scaffold remains in the body. Creating a pre-existing vascular network accelerates overall construct vascularization, as host vessels need only connect to the outer regions, potentially leading to faster graft integration [9,12]. This approach also involves the co-culture of endothelial cells with other cell types, such as osteoblasts, and aims to create capillary-like structures before implantation. In conditions simulating the physiological niche, especially in a three-dimensional environment, endothelial cells naturally organize into tubular networks [13,14].

Prevascular structures formed in vitro can rapidly connect to host vasculature upon implantation, accelerating graft vascularization and survival. This is crucial in bone tissue engineering, where the interplay between bone cells and blood vessels is essential [8,15,16,17]. For effective prevascularization, endothelial cells are typically combined with other cell types. The challenge lies in optimizing culture conditions that support both vascular network formation and tissue development, ensuring the creation of a functional prevascularized construct.

Calcium metaphosphates (CMPs) are a distinct type of bioactive ceramic with a polymeric structure [Ca(PO_3_)_2_] [18]. They have gained significant attention as potential bone substitute materials due to their close resemblance to the calcium phosphate found in bone, despite having a slightly different chemical structure compared to hydroxyapatite (HA). CMP is known to elicit specific biological responses at the material–tissue interface, promoting the formation of a strong bond between the material and surrounding tissue [19].

This study aims to develop a novel 3D porous calcium metaphosphate (CMP) scaffold that supports osteoconduction and vascularization for cranio-maxillofacial reconstruction. By establishing a co-culture model of endothelial cells and osteoblasts, we sought to create a prevascular network within the scaffold, focusing on its fundamental biological interactions and vascularization potential as critical first steps toward developing an effective bone graft material for large, complex bone defects.

## 2. Materials and Methods

### 2.1. Scaffold Fabrication

Porous scaffolds of CMP were produced by mixing commercial monocalcium bis-phosphate monohydrate (Ca (H_2_PO_4_)_2_·H_2_O) (Fluka) and commercial polyvinyl alcohol (PVA) in a 4:1 weight ratio, 0.7 g of the powder mixture was placed in a 12 mm diameter stainless steel mould, and the mixture was pressed at 10 MPa (INSTRON) for 2 min to yield a solid disc before being heated to 900 °C at 10 °C/min and remaining at 900 °C for 6 h before being furnace-cooled to room temperature. Pores were created in the scaffolds by the burning of PVA, the pore-forming agent [20].

### 2.2. Scaffold Characterization

The phase purity of CMP scaffolds was determined by X-ray diffractometry (XRD, JEOL JDX 3530) and Fourier Transform Infrared spectroscopy (FTIR, Perken-Elmer Spectrum one). For morphology studies, the scaffolds were fixed on carbon-coated stubs and then gold-coated before being viewed under an FEI Quanta (Field Emission Gun SEM). Energy-dispersive X-ray analysis (EDX) was also employed to provide information on the chemical composition and respective proportions (At%) of the scaffold. Porosity was determined using both X-ray micro-computed tomography (µ-CT) (Skyscan 1172, Skyscan, Belgium) and mercury intrusion porosimetry.

Water uptake and solubility are key factors influencing the degradation rate of porous CMP materials. To determine the dry weight, HA and CMP samples were dried overnight at 110 °C in a drying oven. After weighing, samples (*n* = 4) were immersed in 3 mL of distilled water and stored at 37 °C for 56 days. At specific intervals, samples were removed, blotted with filter paper to remove surface water, and weighed using a digital balance. The percentage of weight change was calculated using

% Hydration = (Final weight − Initial weight)/Initial weight × 100

After each measurement, the water volume was replenished to 3 mL. This process was repeated throughout this study to monitor changes over time.

### 2.3. Cells and Growth Conditions

#### 2.3.1. Human Alveolar Osteoblast Cells (aHOBs)

The primary aHOBs were isolated (ethics and patient consent approved, 05/Q1803/85) and characterized as previously described [21,22]. Cells were incubated in a humidified atmosphere with 5% CO_2_ at 37 °C. The growth medium used was Dulbecco’s Modified Eagle’s Medium (DMEM; D6046), containing 10% fetal calf serum (FCS), 5% of HEPES, 1% minimal essential medium (MEM), 20 mM L-glutamines, 100 units/mL of penicillin, 0.1 mg/mL of streptomycin, and 15% ascorbate powder (all from Sigma, Leeds, UK). Only cells from passages 6–8 were used in this study.

#### 2.3.2. Human Umbilical Vein Endothelial Cells (HUVECs)

Primary human endothelial cells isolated from the umbilical vein (HUVECs) were purchased from Cascade Biologics^®^ (Life Technologies, Scotland, UK). Cells were resuscitated and cultured in a humidified atmosphere (37 °C, 5% CO_2_) in Medium 200 with the addition of serum growth supplements bFGF (20 ng/mL), EFG (10 ng/mL), and human plasma fibronectin (10 µg/mL) (all from Invitrogen, Morecambe, UK). Only HUVECs from passages 5–8 were used in the experiments.

#### 2.3.3. Human Bone Marrow Mesenchymal Stem Cells (hBMSCs)

Undifferentiated human bone marrow mesenchymal stem cells (hBMSCs) were exploited for their potential role in stimulating endothelial tubular formation in the co-culture system. Briefly, primary human hBMSCs were obtained from fresh bone marrow from human iliac crest aspiration (ethics and patient consent approved, KCH REC REF 08/H0808/10) as described previously [23]. Cells were cultured in Dulbecco’s Modified Eagle’s Medium (DMEM; Special D6429), containing 20% fetal calf serum (FCS), 100 units/mL of penicillin, and 0.1 mg/mL of streptomycin (all from Sigma, Leeds, UK), and 10 ng/mL of fibroblast growth factor (FGF) (Prepotech, London, UK) was also added. Only cells from passages 6–8 were used in this study.

### 2.4. Optimization of VEGF Concentration

The response of HUVEC to different concentrations of VEGF was investigated. The VEGF was loaded at concentrations of 5, 10, 20, 30, 50, and 100 ng/mL. Results were obtained on days 1, 4, and 7 by cell counting and methyl thiazolyl tetrazolium (MTT) cell viability assay. To minimize the serum confounding effect, a medium with a 1% low serum supplement was selected for this study to ensure that the sole effect of VEGF on HUVEC could be observed.

### 2.5. Optimization of Co-Culture Media

To determine the optimal media for co-culture, different ratios of HUVEC to MSC and HOB media were investigated. Equal numbers of cells were seeded in direct contact in a 24-well plate. After 24 h, the media were changed; six different media ratios were tested (Table 1).

The co-culture of HUVEC and aHOB or hBMSC was allowed to proliferate for 21 days. The Alamar Blue assay was conducted on days 2, 5, 7, 14, and 21 to determine cell activity and viability in each of the media ratios. To assess whether each cell type proliferated equally within the co-culture system using the optimum ratio of media, determined previously, cells were labelled with cell trackers to allow the identification of specific cell types for cell counting. The immunofluorescent staining of HUVEC, aHOB, and hBMSC cells was carried out using CellTrackers (Invitrogen, Morecambe, UK). Red (C34552) was used for both aHOB and hBMSC and green (C7025) was used for the HUVECs.

### 2.6. VEGF Release from Co-Culture

To determine the amount of VEGF released in the co-culture system, supernatants of co-culture cells in optimized media were collected on days 4, 7, 14, and 21; particulates were removed by centrifugation; and the supernatant was stored at −80 °C for later analysis. VEGF release from the co-culture was measured using the Quantikine^®^ Human VEGF Immunoassay (R&D systems, Oxfordshire, UK). Optical density was determined using a DYNEX Opsys technologies reader (test wavelength: 450 nm; reference wavelength: 570 nm).

### 2.7. Angiogenesis Assay (Endothelial Tube Formation)

A collagen gel sandwich culture was utilized to visualize tubule formation. The gel was prepared by mixing 0.7 mL of 10× Eagle’s DMEM solution with 6 mL of rat-tail type I collagen, which was dissolved in 0.1 M acetic acid at a protein concentration of 2.035 mg/mL. This mixture was then neutralized using 5 M and 1 M sodium hydroxide. Following the preparation of the gel, 300 μL of the collagen solution was added to each well of a 24-well plate and incubated for 30 min to allow it to set. A 250 µL cell suspension was prepared for three conditions: (A) HUVEC, (B) HUVEC with HOB, and (C) HUVEC with hBMSC. Each suspension (1 × 10⁵ cells/well) was added on top of the set collagen and incubated for 60 min for cell adhesion. Then, 300 µL of collagen was layered over the cells to form a “sandwich” and incubated until set. Following this, 1 mL of medium was added to the wells: HUVEC-specific media for group (A) and optimized co-culture media for groups (B) and (C).

Cultures were allowed to grow for 14 days with microscopic observations every 12 h. The media were changed on day 7 without disturbing the collagen construct using low-power vacuum suction. Endothelial tube morphogenesis was examined by phase contrast microscopy (Olympus IX51, Nagano, Japan) and images were captured using a Colorview II digital camera (Olympus, Nagano, Japan). At the end of the assay, both semi-quantitative CD31 ELISA and tubule area were assessed using a CD31 ELISA & Histology Staining Kit (TCS cell works, Buckingham, UK). Optical density was determined using a DYNEX Opsys technologies reader (test wavelength: 405 nm; reference wavelength: 570 nm). For image analysis, four randomly selected fields of view were photographed in each quadruplicate well per condition. Images were captured using low magnification (×40) and converted to binary format using LCS Leica confocal software (version 2.5, Leica GmbH, Germany). The binary threshold function was adjusted to obtain the best contrast of tubules with the background. Tubule area was calculated as the total number of pixels in threshold-adjusted images and then converted to µm^2^.

### 2.8. Phenotypic Characterization of HUVEC and aHOB

To evaluate the stability of EC and osteoblast phenotype within the co-culture, and to further establish the optimum seeding conditions for tubular formation in the 3D βCMP scaffold, semi-quantitative PCR was performed to verify both EC and osteoblast-specific markers (Table 2). RNA was isolated from a monolayer of aHOB, and the HUVEC co-culture from day 14 was extracted. Reverse transcription was performed using a Thermal Cycler Veriti (Applied Biosystems, UK). The PCR product was loaded onto 1.2% *w*/*v* agarose gel (Bioline, Germany), the gel was viewed by a PC-linked UV transilluminator, and amplified DNA fragments were determined using the DNA ladders.

### 2.9. Three-Dimensional Co-Cultures of HUVEC and aHOB on βCMP Scaffold

Following sterilization of the βCMP scaffolds, cells were seeded at 5 × 10^5^ per scaffold with an equal proportion of HUVEC to aHOB cells. After incubation for 60 min, 400 µL of collagen type I (as described above) was added to each well. Once the collagen was set, 1 mL of co-culture media (40% HOB: 60% HUVEC) with an additional 10 ng/mL of VEGF was added. The medium was changed after 7 days. All incubations were carried out in a humidified atmosphere (37 °C, 5% CO_2_) for 14 days.

### 2.10. Scanning Electron Microscopy for Cell Morphology

On day 14, samples were fixed and processed, and SEM was used to examine the morphology of cells and tubular structures.

### 2.11. Immunocytochemistry

The expression pattern of the endothelial cell adhesion molecules (PECAM-1 or CD31, von Willebrand factor) and structural protein vimentin was examined by the immunofluorescent staining of cellular constructs using a Leica multi-photon 2 confocal microscope (Leica GmbH, Germany) with an acoustic–optical beam-splitter spectral scan head (AOBS) to visualize the tubular networks inside the βCMP scaffold. The dilution factor of each primary and secondary antibody is summarized in Table 3. The tubular formation inside of the porous βCMP scaffold was confirmed by real-time recording for different depths of βCMP, using the 3D time-lapse function of LCS Leica software. The scaffolds were also stained immunohistochemically by both CD31 and vWF to identify the microtubule formation (TCS Cell works, Buckingham, UK).

### 2.12. Statistical Analysis

All cellular experiments were performed in quadruplicates and repeated at least three times, demonstrating reliable reproducibility. Data are expressed as mean ± standard deviation (SD). Potential outliers were examined using the interquartile range (IQR) method, with any identified outliers reported in the results section. Statistical analyses were performed using STATA^®^ statistical software version 17. The significance level was predetermined at *p* < 0.05 for all tests.

The Shapiro–Wilk test was conducted to assess the normality of data distribution. For normally distributed data, one-way analysis of variance (ANOVA) was used to compare means among groups, followed by Tukey’s post hoc test for multiple comparisons. For data not following a normal distribution, the non-parametric Kruskal–Wallis’s test was employed, with the subsequent Dunn’s procedure for pairwise comparisons.

## 3. Results

### 3.1. Characterization of CMP Scaffold

#### 3.1.1. X-Ray Diffraction

**Figure 1:**
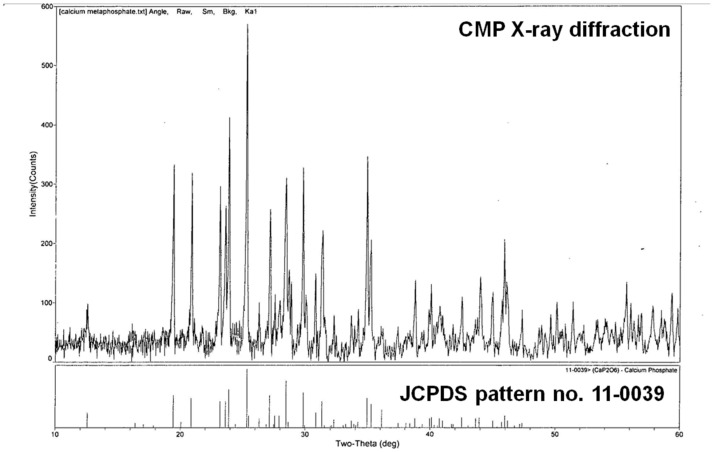
X-ray diffraction patterns for the CMP scaffold showing sharp, high peaks at 25.28°, 28.50°, and 31.75°. The peaks observed correspond to predominant phases of crystalline β-meta calcium phosphate (JCPDS 110039).

The XRD diffraction pattern of the CMP showed sharp diffraction-characteristic peaks that appeared at around 25.28°, 28.50°, and 31.76° (Figure 1). This corresponds to the characteristic peaks of crystalline β-meta calcium phosphate as published earlier [24,25] and by the Joint Committee on Powder Diffraction Standards (JCPDS no. 11-0039).

#### 3.1.2. Infrared Spectroscopy

**Figure 2:**
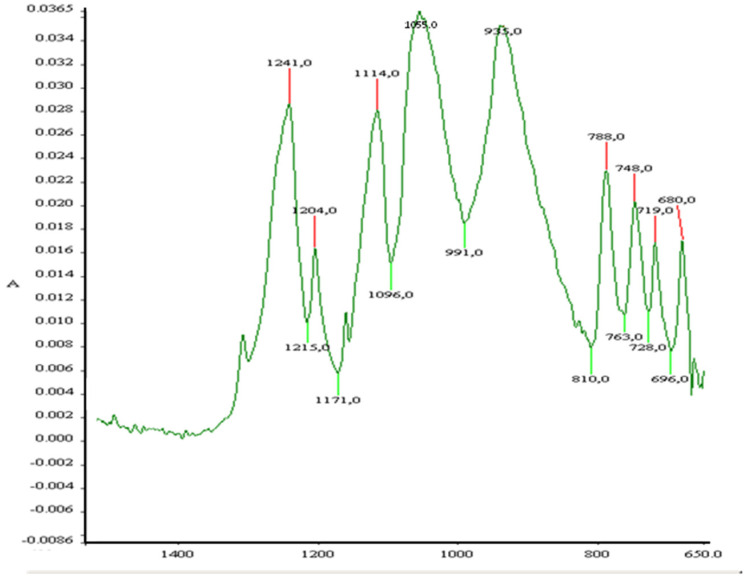
FTIR spectrum of calcium metaphosphate scaffold shows expansion of the fingerprint region.

The infrared spectra of the CMP showed strong characteristic absorption peaks at 1241 cm^–1^, 1114 cm^–1^, 1055 cm^–1^, 935 cm^–1^, and 788 cm^–1^ (Figure 2). These results for the P–O bond and also the PO_3_ group correspond to those reported by Briche et al. [26]. No characteristic peaks of PVA and organic residues were observed in the βCMP scaffold, confirming that they had been obliterated following the sintering process.

#### 3.1.3. Morphology and Pore Structure of CMP Scaffold

**Figure 3:**
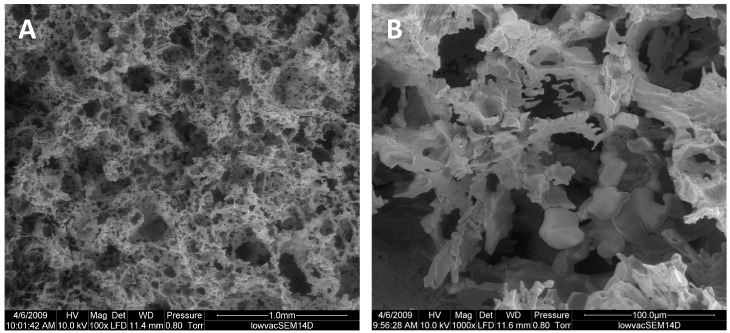
SEM micrographs of porous CMP scaffold: (**A**) ×100 and (**B**) ×1000 viewed under low vacuum at 10.0 kV.

**Figure 4:**
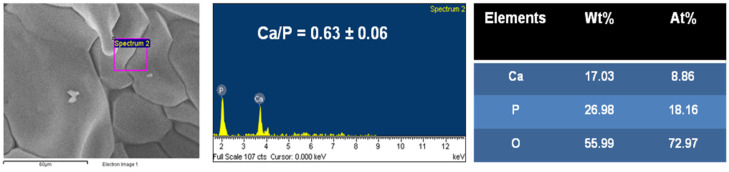
EDS microanalysis of the chemical composition of CMP scaffold and tabulated data on Ca, P, and O elemental proportions by weight (Wt%) and by number of atoms (At%).

The topography of the CMP was observed by SEM (Figure 3A,B) to possess a predominant porosity over a wide range of pore sizes from 80 to 100 µm. In addition, microporosity pores (>400 µm) were also observed across the surface of the scaffold. EDX microanalysis results showed porous CMP with a Ca/P ratio of 0.48 ± 0.046 (*n* = 4) (Figure 4).

**Figure 5:**
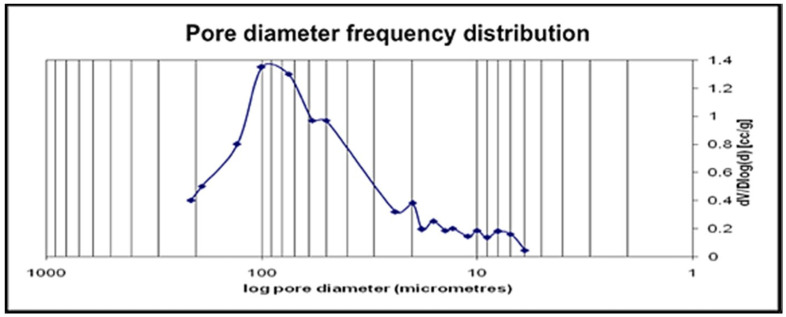
Pore size distribution of CMP obtained from mercury intrusion porosimetry.

The proportion of the number of atoms corresponded to the chemical composition of β-calcium metaphosphate (CaP_2_O_6_). The pore diameter frequency distribution and % porosity data were collected via MIP. Pores were within the 0–217 µm range studied, and 100 µm pores were the most frequent within the structure of CMP (Figure 5), although smaller and larger pores were also detected.

In addition, with the same method, 71% of total porosity was also observed in the CMP scaffold. The actual pore structure of CMP was determined by µ-CT. Rendered 3D scans of the CMP scaffold revealed good interconnectivity from the top to bottom of the construct with an approximate porosity of 68.6%. µCT structural analysis values are shown in Table 4.

The interconnectivity of the porous CMP scaffold was confirmed by using the “flight recorder” function of CTvox software, which allows observation of the internal structures throughout the CMP scaffold.

#### 3.1.4. Water Uptake

**Figure 6:**
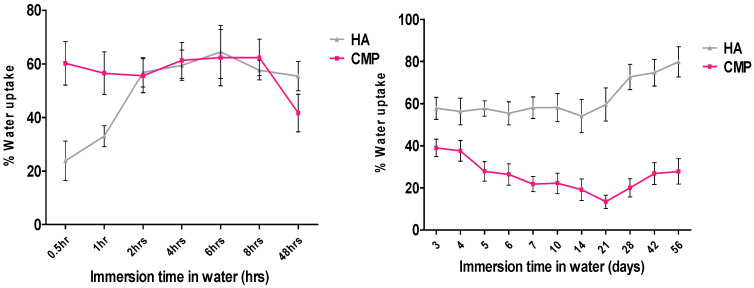
Graph showing % water uptake of HA and CMP at 37 °C cover period of 56 days (time(x) axis not to scale, error bar = SD).

After immersion in distilled water, the weights of HA and CMP were recorded over 56 days (Figure 6). CMP exhibited higher water uptake during the first 2 h compared to HA, which swelled more slowly at the start. Over time, HA showed a gradual increase in water uptake, while CMP displayed a decreasing trend, reaching a final swelling of 30.65% of its initial dry weight after 56 days. Weight fluctuations were observed in both materials throughout this study.

### 3.2. Optimization of VEGF Concentrations

**Figure 7:**
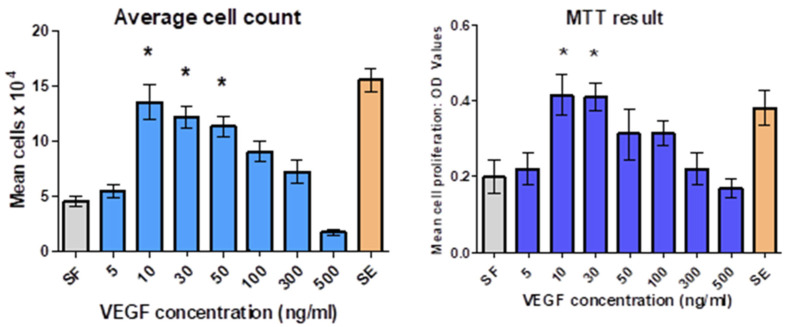
The dose-dependent effect of varying VEGF concentrations on HUVEC proliferation; grey columns represent EC media supplement-free (SF) and brown columns represent EC media with supplements (SE). Data presented as mean ± standard deviation (SD) from quadruplicates and three independent experiments. One-way analysis of variance (ANOVA) was used to compare the means among groups; * *p* < 0.05 was considered statistically significant.

Results were obtained using cell counts and the MTT assay (Figure 7).

The average cell number obtained for days 1, 4, and 7 indicated that high doses of VEGF (500 ng/mL) downregulated HUVEC proliferation, with the optimum stimulatory dose found to be between 10 and 30 ng/mL. Statistical analysis revealed a significant difference between optimum doses and serum-free (*p* < 0.05). In addition, it was evident from both the cell counts and MTT assay that 5 ng/mL of VEGF was found to not affect cell proliferation.

### 3.3. Optimization of Co-Culture Media

**Figure 8:**
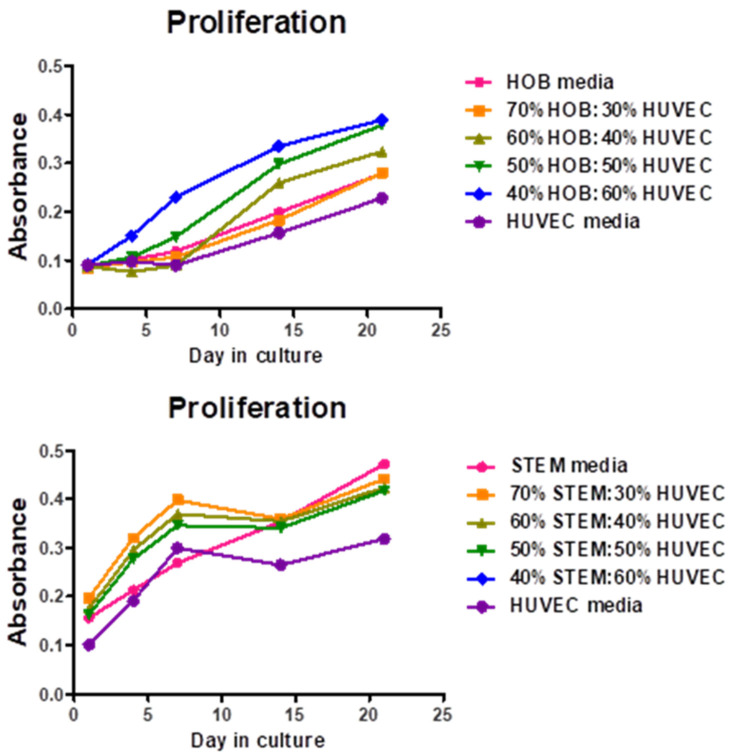
AlamarBlue cell proliferation assay over 21 days with various ratios of co-culture media of aHOB and HUVEC. The highest cell growth was found in the 60%HUVEC:40%HOB ratio. Statistical analysis revealed a significant difference between optimum doses and serum-free (*p* < 0.05).

To determine the ideal medium for the maximum proliferation of cells within the co-culture system, different ratios were investigated. Over 21 days, an increasing trend was observed universally. The ratio of 60% HUVEC: 40% HOB media was optimum for HUVEC/HOB co-culture and 30% HUVEC: 70% MSC for HUVEC/hBMSC co-culture (Figure 8).

**Figure 9:**
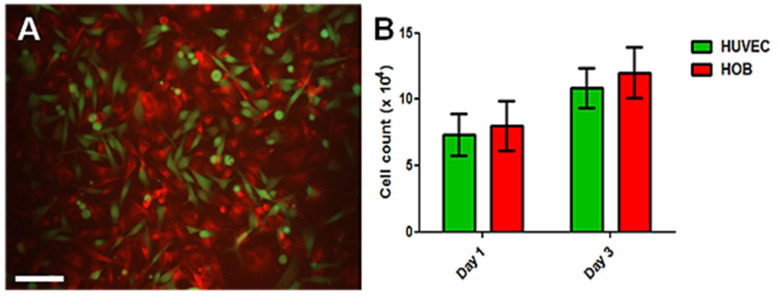
(**A**) Fluorescent micrographs showing co-cultures of alveolar osteoblast cells (aHOBs) in red and umbilical vein endothelial cells (HUVECs) in green, stained with CellTracker™ (scale bar = 150 µm); (**B**) no significant difference was observed between the numbers of each cell type on either day 1 or day 3, with *p*-values of *p* = 0.79 and *p* = 0.64 for days 1 and 3, respectively. Results are expressed as the mean value ± standard deviation (SD), derived from four replicate samples across three separate experimental runs.

In addition, the growth of each cell type was also confirmed using immunofluorescent tracking and cell counts were confirmed using fluorescent microscopy (Figure 9A,B).

### 3.4. VEGF Release from Co-Culture

**Figure 10:**
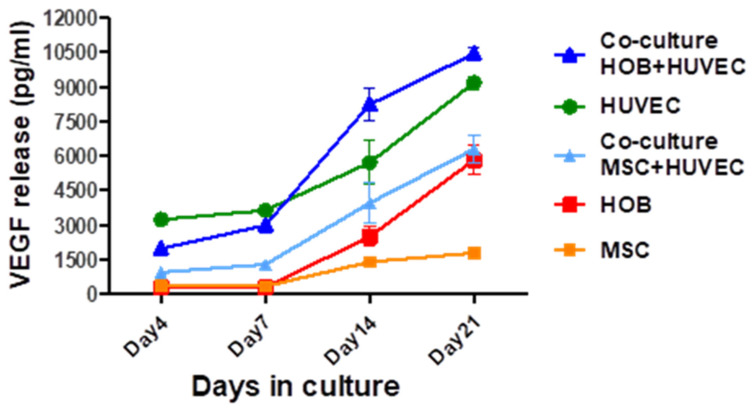
The amounts of VEGF released in co-culture (aHOB/HUVEC and hBMSC/HUVEC) and in monoculture for HUVEC, aHOB, and hBMSC. The data are presented as the average ± standard deviation (SD), calculated from quadruplicate samples obtained from three independent experimental iterations. The ratio of 60% HUVEC: 40% HOB media was optimum for HUVEC/HOB co-culture (*p* < 0.001) and 30% HUVEC: 70% MSC for HUVEC/hBMSC co-culture (*p* < 0.05). Data were analyzed using Stata^TM^ statistical software. One-way analysis of variance (ANOVA) was used to compare the means among groups, and non-parametric Kruskal–Wallis analysis was used where the data were not normally distributed.

Before adding the exogenous VEGF to the cells in co-culture to examine the effect on tubular formation, the amount of intrinsic VEGF released from each cell type and in the co-cultured cells was investigated. Figure 10 shows that VEGF release increased with time.

The highest VEGF release was observed in the co-culture of aHOB/HUVEC up to 10,455.6 pg/mL on day 21 followed by HUVEC monoculture. In contrast, very low levels of VEGF were released by hBMSC in monoculture. Both the co-culture of aHOB/HUVEC and hBMSC/HUVEC released more VEGF than the monoculture of aHOB or hBMSC alone (Figure 10).

### 3.5. Angiogenesis Assay

**Figure 11:**
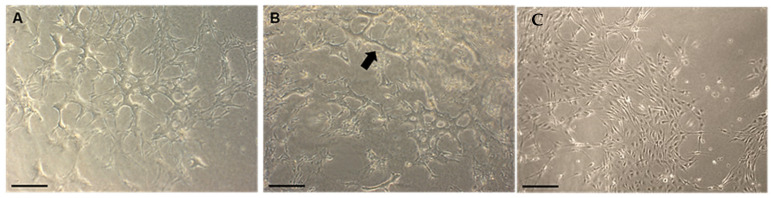
(**A**) Phase contrast micrographs showing a greater number of networks of branching and anastomosing cords of cells at day 10; (**B**) a longer tube length (arrow) was observed in the co-culture of HUVEC/HOB with VEGF; (**C**) Co-culture of HUVEC/hBMSC (Scale bar = 200 µm).

HUVECs in all culture conditions, except for the co-culture with hBMSC (with and without VEGF), appeared more elongated and organized to form aggregates. By day 10, a more organized tubular-like network formation was evident. Interestingly, co-cultures of HUVEC and aHOB with VEGF formed tubule networks that contained more total junctions and longer tubules (Figure 11A,B). In contrast, for the co-culture of HUVEC and hBMSC, no concomitant cell aggregates or lumenization of the nascent capillary-like structure was observed (Figure 11C).

**Figure 12:**
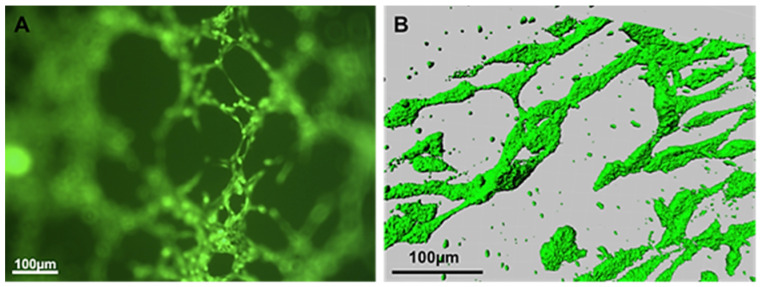
(**A**) Fluorescent micrograph of tubular structures of HUVEC stained with CD31 antibody. (**B**) Reconstructed image of HUVEC using LCS Leica confocal software (version 2.5).

At the end of the experiment, quantification of angiogenesis was measured using CD31/PECAM-1 ELISA and image analysis. The emergent tubular structures were visualized with CD31 staining. The area occupied by aggregates of HUVEC was reconstructed using image software (Figure 12A,B).

**Figure 13:**
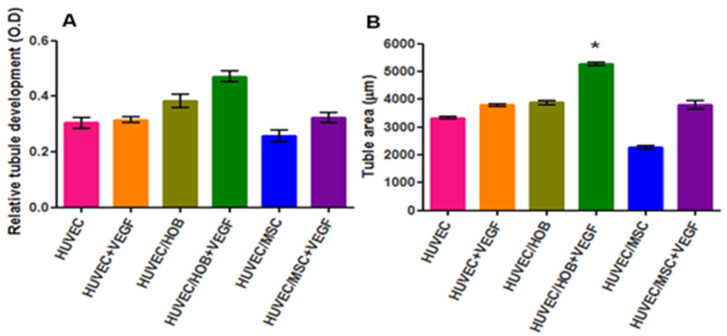
Comparison of the relative amount of tubular formation in different culture systems using (**A**) CD31 ELISA and (**B**) image analysis software. Data presented as mean ± standard deviation (SD) from quadruplicates obtained from triple-repeated experiments. One-way analysis of variance (ANOVA) indicated a significant difference among the culture groups (*p* < 0.01). Tukey’s post hoc test showed that the mean values of the co-cultures of HUVEC/aHOB + VEGF were statistically significantly higher than co-cultures of HUVEC/hBMSCs (* *p* < 0.01).

The results obtained from CD31 ELISA confirmed a degree of tubule development in the wells. These showed that tubular formation in collagen gel was greatly enhanced by the co-culture of HUVEC and aHOB in the presence of VEGF (Figure 13A). An additional role of VEGF was observed; the results showed a greater effect in co-culture than monoculture when VEGF was added to the respective culture media. Furthermore, the total area of tubules (Figure 13B) was similar to the ELISA results, with statistical significance (*p* < 0.001) in co-cultures of HUVEC/aHOB + VEGF when compared to HUVEC/hBMSCs.

### 3.6. Phenotypic Characteristics of Co-Culture of HUVEC and aHOB

**Figure 14:**
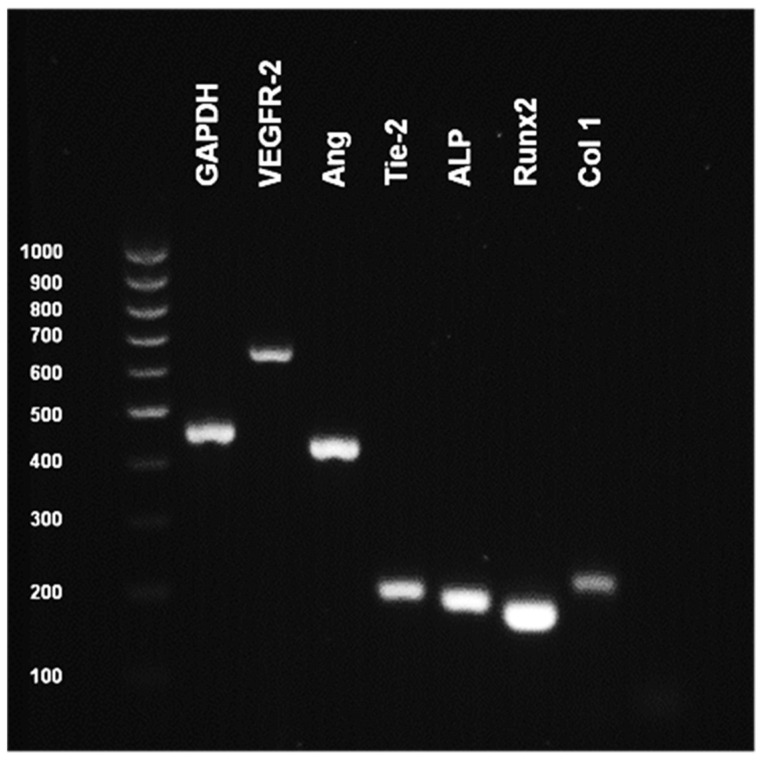
Reverse-transcriptase polymerase chain reaction (RT-PCR) for endothelial and osteoblast marker expression from selected co-culture conditions (HUVEC/aHOB+VEGF) after 14 days. Endothelial cell markers (VEGFR-2, Angiopoietin-1, Tie-2) and osteoblast markers (ALP, Runx2, col1) observed in cell lysates of co-culture confirmed the phenotypic expression of endothelial cells.

The co-culture of HUVEC and aHOB in the presence of 10 ng/mL of VEGF demonstrated ideal conditions for initiating in vitro prevascularization of the CMP scaffold. The ability of the cells to retain phenotypic characteristics in the experimental conditions was confirmed by RT-PCR. The results for PCR showed both EC (VEGFR-2, Angiopoietin1, and Tie-2) and osteoblast (ALP, Runx2, and Col1) mRNA to be present in co-culture cell lysates (Figure 14). Although these results were only qualitative, they confirmed the expression of endothelial and osteoblast cell phenotypic markers for up to 14 days in co-culture.

### 3.7. Prevascularization of 3D βCMP Scaffold

**Figure 15:**
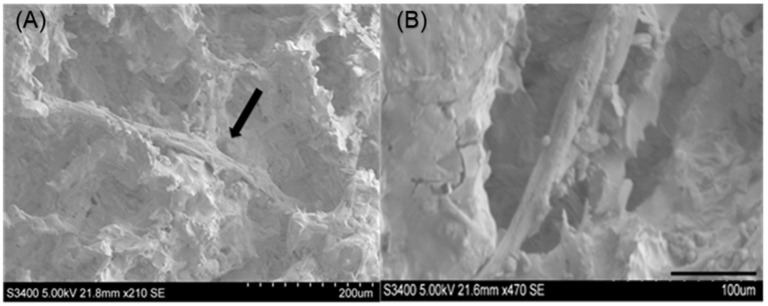
(**A**) The SEM image shows the tube-like structure across the surface of CMP (arrow). (**B**) A tubular structure inside the calcium metaphosphate (CMP) scaffold.

After 14 days of an ideal co-culture system, the outgrowth of co-culture cells adhering to the surface and also inside of the porous CMP scaffold was evident. SEM revealed tube-like structures across the surface (Figure 15A) and inside of the CMP scaffold (Figure 15B).

**Figure 16:**
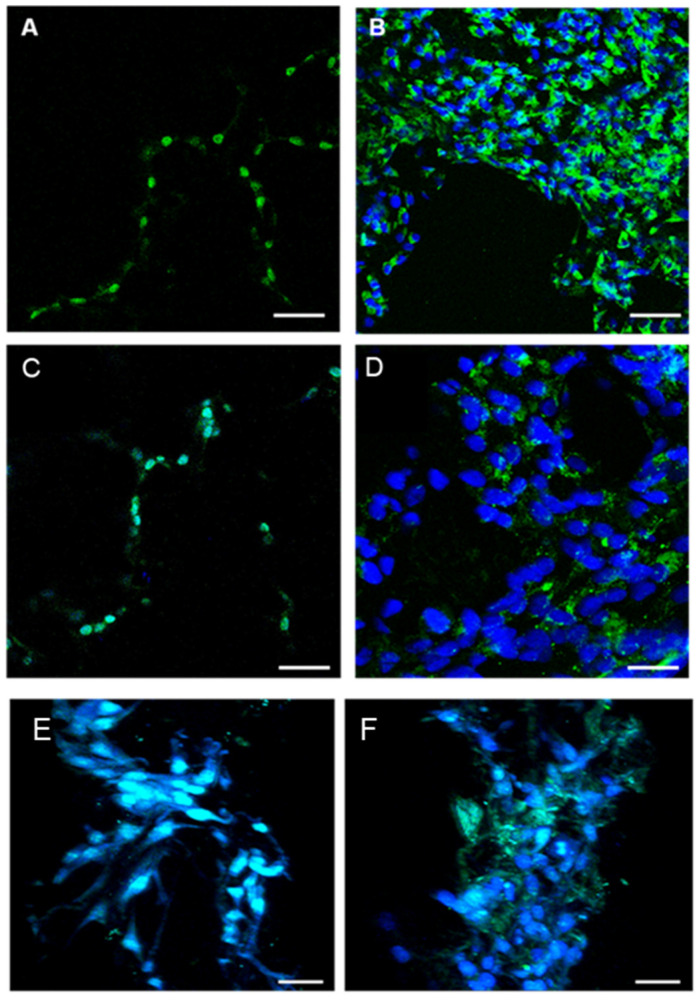
(**A**) Immunofluorescence staining of CD31, EC marker, inside the pore structure of CMP, and (**B**) image from the outside looking in through half of the fractured CMP (scale bar = 100 µm). (**C**) Immunofluorescent staining for vWF, EC marker, inside the pore structure of CMP, and (**D**) image from the outside looking in through the fractured CMP (scale bar = 100 µm). Immunofluorescent staining for (**E**) actin and (**F**) vimentin filaments inside the 3D scaffold (scale bar = 100 µm).

Immunofluorescent staining of EC with CD31 (Figure 16A,B), vWF (Figure 16C,D), and actin and vimentin (Figure 16E,F) demonstrated the organization of cells forming capillary-like structures inside the porous CMP scaffold.

Each EC marker showed the organization of cells forming capillary-like structures inside the porous CMP. This was further confirmed by capturing images at different depths of the scaffold (Supplementary Materials). This animation confirmed that cells were able to infiltrate the interconnecting pores of the scaffold and arrange themselves in an orderly manner to form tubule-like structures.

**Figure 17:**
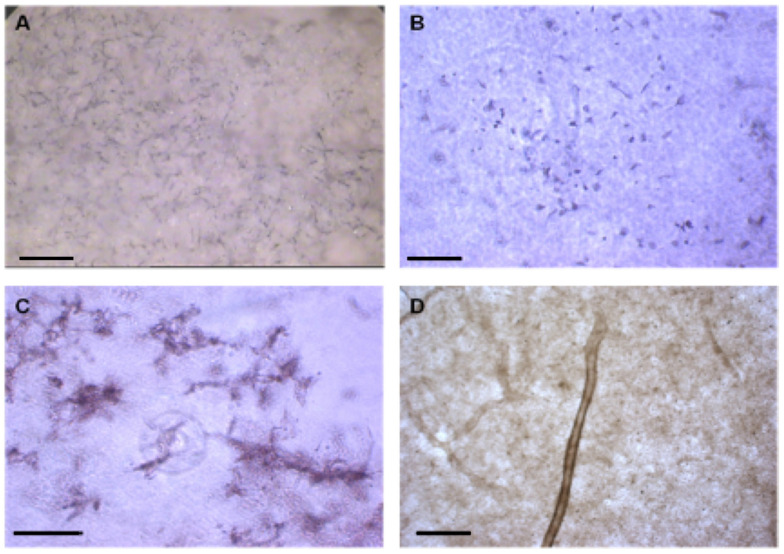
Immunohistochemical staining of (**A**) CD31 on CMP (10× magnifications), (**B**,**C**) CD31-positive ECs, and (**D**) VWF staining of a tubular structure in porous CMP (40× magnifications) (scale bar = 100 µm).

The qualitative immunohistochemistry of EC markers in co-culture showed that cells were evenly distributed throughout the porous surface of the CMP scaffold (Figure 17A). Distinct differences in CD31^+^ cells were observed between the monolayer (Figure 17B) and in 3D CMP, which were organized into capillary-like networks (Figure 17C). In addition, tubular-like structures with branches were confirmed by vWF staining as viewed by non-inverted microscopy (Figure 17D).

## 4. Discussion

Our study aimed to develop a cell-seeded scaffold for a prevascularized bone graft to address insufficient vascularization in large bone defects. While composite materials like polycaprolactone (SPLC) [27] or silk fibroin (SF) [28] have been used, calcium phosphate-based materials are preferred due to their similarity to natural bone, non-toxicity, biocompatibility, and osteoconductive properties [19]. Various synthesis methods for calcium phosphate bioceramics include co-precipitation and sintering. The sintering process, including heating rate, temperature, and duration, is crucial for achieving the desired properties. βCMP was formed by heating MCP to 900 °C to form the crystalline. PVA acted as a pore-forming agent during sintering, creating a highly porous microstructure through the release of water and carbon dioxide [20].

The FTIR, XRD, and EDX data confirmed that the chemical composition of both scaffolds was crystalline β-calcium metaphosphate (β-Ca (PO_3_)_2_). Low-vacuum SEM revealed a porous βCMP topography with mixed pore sizes. MIP and µCT analysis confirmed a wide range of pore sizes from macro- (>400 μm) to micro (<80 μm)-porosity, aligning with the optimal range for bone tissue engineering (100–500 µm) [11], while the optimal pore size for bone ingrowth was claimed to be 100–400 µm [29]. Larger pores facilitate cellular migration and proliferation, while micropores support nutrient, gas, and ion exchange. Three-dimensional modelling using CTvol software (skyscan, Kontich, Belgium) demonstrated good pore interconnectivity, promoting cell infiltration and colonization. The scaffold’s pore structure, with the highest frequency between 80 and 100 µm, effectively balances mechanical strength with biological requirements for cell infiltration and vascularization, making it suitable for bone tissue engineering applications.

The total porosity of βCMP, measured by MIP (71%) and µCT analysis (68.3%), falls within the appropriate range for bone tissue engineering application as the natural bone porosity ranges from 50 to 90% [11]. While increasing porosity typically reduces the mechanical properties, this study prioritizes creating a material that allows nutrient ingress and cell migration. The focus was on maintaining an average porosity of 71%, with emphasis on pore size and interconnectivity.

Calcium metaphosphate (CMP) scaffolds exhibit a higher water uptake compared to hydroxyapatite (HA), indicating a more porous structure and faster degradation rate. This characteristic suggests that CMP may have compromised mechanical properties, making it less suitable for load-bearing applications. However, the increased porosity and degradation rate of CMP can be advantageous in non-load-bearing scenarios where gradual material resorption is desired, as it facilitates tissue integration and remodelling.

MTT assay and cell counts showed that the optimal VEGF dose for HUVEC was 10–30 ng/mL, with 5 ng/mL being ineffective. Therefore, 10 ng/mL was selected as the lowest effective dose, aligning with several studies [30,31,32]. In contrast, Silva and Mooney (2010) found 30 ng/mL optimal for human microvascular dermal endothelial cells (HDMECs), highlighting cell-specific responses. In vivo, GF levels are tightly regulated, as excess VEGF can cause abnormal vessel formation [33]. Determining the minimum effective concentration is crucial to achieve optimal results without side-effects like receptor downregulation.

Various studies have explored co-culture conditions for endothelial cells (ECs) and bone cells. This study found optimal media ratios of 60% HUVEC to 40% HOB media, and 30% HUVEC to 70% MSC media for maximal cell proliferation. Cell trackers confirmed equal growth and survival of both cell types. Achieving similar proliferation rates and maintaining phenotypes of both cell types are crucial for successful co-culture systems. The ideal cell ratio for vasculogenesis in vitro is debated. Unger et al. suggested a ratio of 5:1 of the endothelial cells to osteoblasts [34], while another study found that lower percentages of HUVECs stimulated prevascular network formation [9]. Our study demonstrated equal growth of HUVECs and aHOBs in co-culture. RT-PCR analysis of osteoblasts (ALP, RUNX2, Col1) and EC (VEGFR-2, Ang, Tie2) markers confirmed the maintenance of phenotypic expression in co-culture.

Collagen matrices allowed direct observation of cell organization and microcapillary-like tube formation, resembling the natural ECM for vessel formation [35]. The vasculogenic process was influenced by cell type and exogenous VEGF. HUVEC-hBMSC co-cultures in collagen gel proliferated but did not form networks without VEGF after 14 days. However, HUVEC-aHOB co-cultures and the HUVEC monoculture with VEGF formed tubule-like structures, consistent with Donovan D’s findings [36]. The EC–osteoblast relationship enhanced network formation without exogenous VEGF through crosstalk and support. VEGF release experiments showed that HUVEC-aHOB co-cultures produced intrinsic VEGF up to 10,455.6 pg/m, near the optimal dose of VEGF (10 ng/mL).

The collagen scaffold provides a more physiological environment for cells, mimicking the natural ECM [37,38]. Collagen promotes cell migration, including EC, and EC binding to the ECM via integrins is crucial for VEGF-induced angiogenesis [39]. Unlike other biomaterials, βCMP demonstrated HUVEC adhesion, orientation, and microcapillary-like tube formation without surface modification in both monoculture and co-culture. This contrasts with studies requiring fibronectin coating for HUVEC adhesion, proliferation, and survival [34]. βCMP’s success may be due to its properties and culture conditions. Unlike polymeric scaffolds, βCMP promotes cellular interaction with adsorbed proteins (including fibronectin) and media-supplied nutrients and growth factors.

Our study has several limitations that should be acknowledged. Primarily, the study predominantly utilizes human umbilical vein endothelial cells (HUVECs) for the endothelial component of the co-culture. Although HUVECs are a common model for endothelial cells, they may not accurately reflect the behaviour of endothelial cells found in bone tissue. Employing bone-specific endothelial cells could yield more relevant insights for bone tissue engineering applications. Furthermore, this study does not evaluate the long-term performance of the prevascularized scaffold or its degradation profile in physiological conditions, both of which are critical for understanding its behaviour in vivo. It is also important to note that our study does not cover the mechanical properties of the scaffold, which are crucial for bone tissue engineering applications. These limitations highlight the need for further research, which will focus on refining this approach to maximize its clinical applications, aiming to bridge the gap between scaffold implantation and successful vascular integration in complex bone defects.

## 5. Conclusions

This study demonstrates that the calcium metaphosphate (CMP) scaffold exhibits promising structural and biological properties for bone tissue engineering, particularly in cranio-maxillofacial reconstruction. By co-culturing osteoblasts and endothelial cells, we successfully established a prevascularized network within the scaffold, addressing the critical challenge of insufficient vascularization in large bone defects. The scaffold’s porosity, interconnectivity, and ability to promote cell infiltration and capillary-like structure formation make it a strong candidate for further investigation. Future research will focus on refining this approach to enhance clinical applications by bridging the gap between scaffold implantation and successful vascular integration in complex bone defects. Additionally, future studies will include mechanical testing, such as compressive strength and fatigue resistance, to ensure the scaffold’s suitability for load-bearing applications. Together, these efforts aim to improve the efficacy of synthetic bone grafts, leading to faster healing and better functional outcomes, particularly in maxillofacial and large bone reconstructions.

## Figures and Tables

**Table 1:** Different ratios of culture media used in co-culture proliferation assay.

HOB or hBMSC Medium (%)	HUVEC Medium (%)
100	0
70	30
60	40
50	50
40	60
0	100

**Table 2:** Primer’s nucleotide sequences and expected PCR product sizes.

Target Gene	Sequence (5’→3’)	Product Size
GAPDH		
Forward Reverse	ACC ACA GTC CAT GCC ATC AC TCC ACC ACC CTG TTG CTG TA	452 bp
VEGFR-2		
Forward Reverse	GTG ACC AAC ATG GAG TCG TG CCA GAG ATT CCA TGC CAC TT	660 bp
Angiopoietin 1		
Forward Reverse	AGA GGC ACG GAA GGA GTG TG CTA TCT CCA GCA TGG TAG CCG	410 bp
Tie 2		
Forward Reverse	CGA GTT CGA GGA GAG GCA ATC TCA GGT ACT TCA TGC CGG G	148 bp
ALP		
Forward Reverse	CCA CGT CTT CAC ATT TGG TG AGA CTG CGC CTG GTA GTT GT	196 bp
Runx2		
Forward Reverse	CAG ACC AGC AGC ACT CCA TA CAG CGT CAA CAC CAT CAT TC	178 bp
Col1		
Forward Reverse	CCA AAT CTG TCT CCC CAG A TCA AAA ACG AAG GGG AGA T	213 bp

**Table 3:** Dilutions of fluorescent stains for primary and secondary antibodies.

Antibodies	Dilution Used	
	Primary	Secondary
PECAM-1, CD31	1:50	1:1000
Von Willebrand (vWF)	1:8000	1:1000
Phalloidin (direct staining)	1:100	
Vimentin	1:100	1:1000

**Table 4:** Micro-CT analysis of porous CMP scaffold.

Description	Abbreviation	Value	Unit
Structure thickness	St.Th	0.0353	Mm
Structure separation	St.Sp	0.072	Mm
Number of objects	Obj.N	4675	
Number of closed pores	Po.N(cl)	336	
Volume of closed pores	Po.V(cl)	0.000256	mm^3^
Surface of closed pores	Po.S(cl)	0.14	mm^2^
Closed porosity	Po(cl)	0.00769	%
Open porosity	PO(op)	68.93	%
Total volume of pore space	Po.V(tot)	7.28	mm^3^
Total porosity	Po(tot)	68.6	%
Connectivity	Conn	42748	
Connectivity density	Conn.Dn	4030	1/mm^3^

## Data Availability

The original contributions presented in the study are included in the article/Supplementary Materials. Further inquiries can be directed to the corresponding author.

## Supplementary Materials

The following supporting information can be downloaded at: https://www.mdpi.com/article/10.3390/jfb16010018/s1, Figure S1. The process of CMP fabrication. (A) The commercial powders of MCP and PVA were used in a 4:1 ratio by weight, and (B) mixed and homogenized by using a porcelain pestle and mortar. (C) The mixture was divided into 0.8 g samples and put into a stainless steel mould before being pressed at 10 MPa. (D) The solid discs were obtained after pressing in an Instron machine. (E) The sintering temperature was 8 °C/minute until 900 °C was reached, and then kept at 900 °C for 6 h, before allowing to cool down. (F) The sintered CMP discs were polished and soaked with PBS for 24 h before sterilization with gamma radiation. Figure S2. Overview of in vitro prevascularization model. aHOB or hBMSCs were co-cultured with HUVEC in specially conditioned media and culture conditions. Tubular formation in the presence of VEGF was assessed using collagen gel. The optimum condition was then applied to the cell-seeded scaffolds to allow a vascularized network formation inside of the CMP construct. Table S1. Plate layout tubular formation assay with or without GF supplements.
