# Supplementary material for: Development of Prevascularized Synthetic Block Graft for Maxillofacial Reconstruction
Source: J Funct Biomater. 2025 Jan 9;16(1):18. doi: 10.3390/jfb16010018 (PMC11766148; doi:10.3390/jfb16010018)
Supplement: Supplementary file 1 [file jfb-16-00018-s001.zip › jfb-3318975-supplementary.pdf]

Supplementary Materials

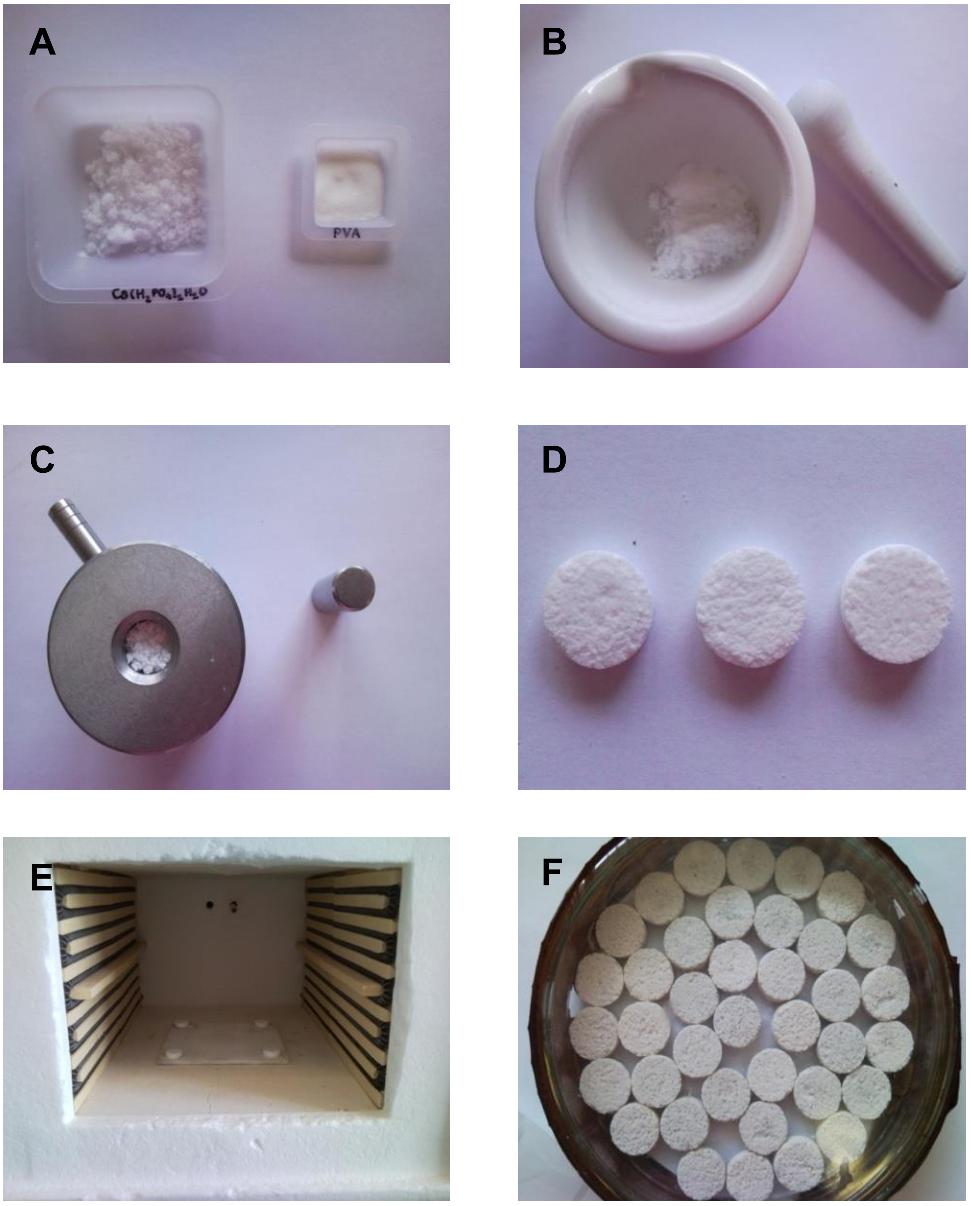

Figure S1 The process of CMP fabrication A) The commercial powders of MCP and PVA were used in a 4:1 ratio by weight, B) mixed and homogenised by using a porcelain pestle and mortar, C) The mixture was divided into 0.8g samples and put into the stainless-steel mould before pressed at 10 MPa. D) The solid discs were obtained after pressing in an Instron machine, E) The sintering temperature was 8 °C/minute until 900 °C was reached, kept at 900 °C for 6 hours before allowing to cool down. F) The sintered CMP discs were polished and soaked with PBS for 24 hours before sterilisation with gamma radiation.

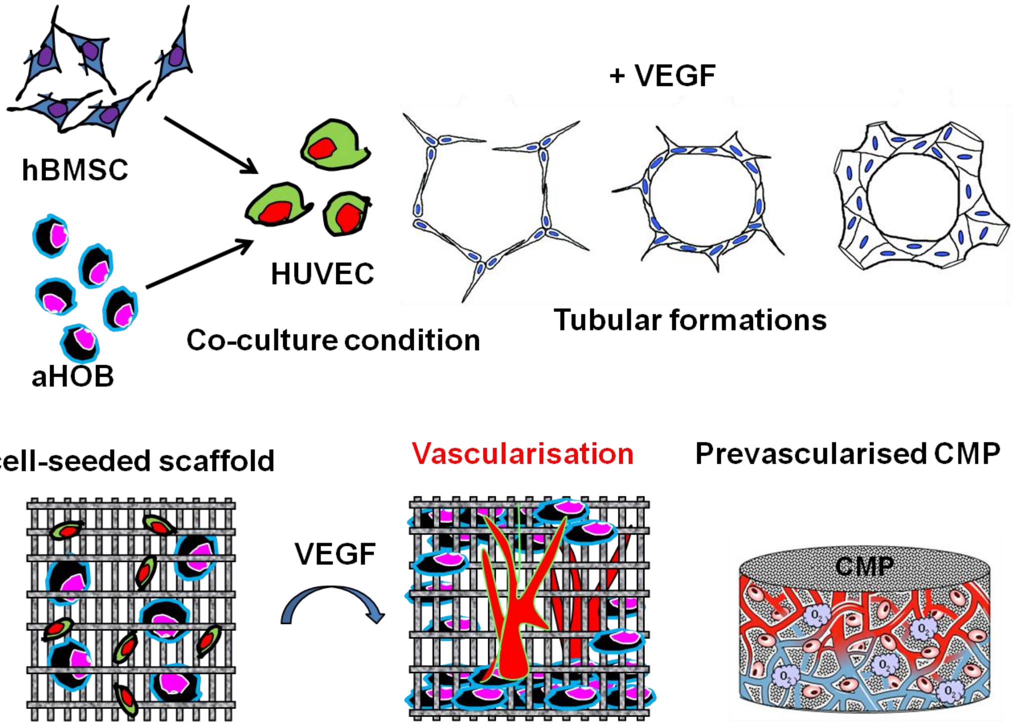

Figure S2 Overview of *in vitro* prevascularisation model, aHOB or hBMSCs were co-cultured with HUVEC in specially conditioned media and culture conditions. Tubular formation in the presence of VEGF was assessed using collagen gel. The optimum condition was then applied to the cell-seeded scaffolds to allow a vascularised network formation inside of the CMP construct.

Table S1 Plate layout tubular formation assay with or without GF supplements

HUVEC	HUVEC +VEGF	HUVEC/HOB	HUVEC/HOB +VEGF	HUVEC/MSC	HUVEC/MSC +VEGF
HUVEC	HUVEC +VEGF	HUVEC/HOB	HUVEC/HOB +VEGF	HUVEC/MSC	HUVEC/MSC +VEGF
HUVEC	HUVEC +VEGF	HUVEC/HOB	HUVEC/HOB +VEGF	HUVEC/MSC	HUVEC/MSC +VEGF
HUVEC	HUVEC +VEGF	HUVEC/HOB	HUVEC/HOB +VEGF	HUVEC/MSC	HUVEC/MSC +VEGF
